# Adherence to immunomodulatory drugs in patients with multiple myeloma

**DOI:** 10.1371/journal.pone.0214446

**Published:** 2019-03-27

**Authors:** Amélie Cransac, Serge Aho, Marie-Lorraine Chretien, Maurice Giroud, Denis Caillot, Mathieu Boulin

**Affiliations:** 1 Department of Pharmacy, Dijon University Hospital and LNC-UMR1231, University of Burgundy & Franche Comté, Dijon, France; 2 Hospital Epidemiology and Infection Control Department, University Hospital, Dijon, France; 3 Department of Clinical Hematology, University Hospital and SAPHIIR-UMR 1231, University of Burgundy & Franche Comté, Dijon, France; 4 Department of Neurology, Dijon University Hospital and LNC-UMR1231, University of Burgundy & Franche Comté, Dijon, France; 5 Department of Clinical Hematology, University Hospital, Dijon, France; 6 Department of Pharmacy, Dijon University Hospital and EPICAD LNC-UMR1231, University of Burgundy & Franche Comté, Dijon, France; University of Milano-Bicocca, ITALY

## Abstract

**Background:**

Immunomodulatory drugs (thalidomide, lenalidomide and pomalidomide; IMID) are widely used in the treatment of multiple myeloma patients. To date, few data are available on IMID adherence in multiple myeloma patients. The aim of our study was to evaluate IMID adherence and to compare two indirect methods to measure IMID adherence in multiple myeloma patients: a specific questionnaire and the medication possession ratio (MPR). Another aim was to explore this specific questionnaire for the assessment of IMID adherence in multiple myeloma patients.

**Methods:**

All consecutive multiple myeloma patients, with at least two consecutive dispensations of thalidomide, lenalidomide or pomalidomide in our hospital were included in this prospective study. IMID adherence was measured using a specific questionnaire and the medication possession ratio. Relationship between the questionnaire scores and variables of interest was evaluated by multiple linear regression with a robust variance estimator.

**Findings:**

Sixty-three patients were included in our study. The mean questionnaire score was 8.2±1.2 and the mean medication possession ratio value was 0.97±0.06. A total of 76% of patients were considered adherent according to the questionnaire (*i*.*e*. score ≥ 8), 94% according to the medication possession ratio (*i*.*e*. MPR ≥ 0.90), and 70% according to the questionnaire and the medication possession ratio. No statistically significant linear association was observed between the questionnaire score and any variables of interest including medication possession ratio. All Cronbach’s alpha were relatively low (range 0.0342–0.2443), showing a low correlation of the different questions with the questionnaire score.

**Conclusions:**

Our study is the first prospective study evaluating IMID adherence in multiple myeloma patients in real life. The high adherence to IMIDs reported here, regardless of the drug, is encouraging considering the efficacy, toxicity and elevated cost of IMIDs. The specific questionnaire should be used with caution to evaluate IMID adherence.

## Introduction

Multiple myeloma (MM) is the second most frequent hematological malignancy after non-Hodgkin lymphoma. It accounted for 1.8% of all new cancer cases and about 10% of all hematological malignancies in 2017 [1–3]. Thalidomide, lenalidomide and pomalidomide belong to the immunomodulatory drug family (IMID). These expensive oral agents are widely used in MM patients because of their demonstrated efficacy in different lines of MM treatment [4]. A European study reported that lenalidomide was the most commonly used agent in first line maintenance therapy in MM [5].

Adherence to long-term therapy for chronic illnesses in developed countries is close to 50% [6]. Concerning oral anticancer agents, Bassan *et al*. reported adherence rates ranging from 40% to 100% [7]. Poor adherence not only affects the patient’s health, but it also puts financial strain on the healthcare system [8, 9]. To our knowledge, there is little data on IMID adherence in MM patients. Only one study evaluated IMID adherence using the Medication Possession Ratio (MPR) in 6731 American MM patients between 2011 and 2013 [10].

The existing methods for measuring medication adherence are direct or indirect, and there is no method considered as the “gold standard” [11]. The direct methods are mostly based on the measurement of the level of medicine or metabolite in blood or urine; they are very difficult or impossible to implement in current daily practice. Indirect methods are mostly based on the analysis of administrative claims (prescription, rate of prescription refills). Administrative data are relatively inexpensive, non invasive, and easy-to-obtain. Even if the MPR is considered a ubiquitous and central measurement for adherence [12], there is no consensus about the best method/tool to measure adherence, each one having limits either of feasibility or reliability.

Thus, the aim of our study was to evaluate IMID adherence in MM patients and to compare two indirect methods for measuring IMID adherence: a specific questionnaire and the MPR. Another aim was to explore this specific questionnaire for the assessment of IMID adherence in MM patients.

## Materials and methods

### Patients

All consecutive MM patients followed in the Hematology Department of our University Hospital with at least two consecutive dispensations of IMIDs (thalidomide, lenalidomide and pomalidomide) between March 1, 2016 and May 15, 2016 were included in our prospective study. The Comité de Protection des Personnes waived the requirement for Ethics Committee approval. The written consent of the patients was obtained before their inclusion.

### Adherence

Medication adherence was measured by two different indirect methods: a specific questionnaire and the MPR.

#### Questionnaire

We used a cancer-specific questionnaire to measure patient adherence to IMIDs. The questionnaire has been previously validated in patients with Chronic Myeloid Leukemia (CML) [13]. It included 10 questions; a “no” response to each question was worth 1 point, except for question 7 where a “yes” response was worth 1 point (maximum score: 10 points). Non-adherence was defined as a score below 8 points [13]. The questionnaire was translated into French by two persons (AC and BS). The French questionnaire was the result of a consensus between the two persons. It was finally revised by a bilingual person (PB).

After consent, the questionnaire was filled in during a pharmaceutical interview. The interview was performed by two pharmacists: the first pharmacist read the questions and explained them further if necessary and the second collected the answers. The time required to complete the questionnaire was also assessed. During the interview, patients were asked if they used specific tools or relied on their caregivers to help them with their intake of IMIDs and if they had treatment-related adverse events.

#### Medication possession ratio

Adherence was also estimated by the measure of the MPR from hospital dispensing data. In France, agents belonging to the class of IMIDs are dispensed by hospital pharmacists. At least two successive dispensations were required to calculate the MPR. The MPR is a ratio between the number of days’ supply within a time interval. It was calculated as the total days’ supply of IMIDs dispensed divided by the number of days between the first dispensation and the end of the last dispensation. The MPR was calculated on a fixed day: the day that the questionnaire was filled in. The MPR cut-off point was 90%. Therefore, a non-adherent patient was identified when the MPR was below 90% [13]. To use MPR as accurately as possible, we took into account the temporary discontinuation of IMIDs. More specifically, we consulted and analyzed all medical data from the medical files for each one of the patients. If a physician had decided to discontinue treatment (for a medical reason such as an episode of febrile neutropenia) the duration was integrated as days of non-treatment in the calculation of the MPR (*i*.*e*. in the denominator).

Data for demographic and clinical characteristics were collected: gender, age, IMID type, International Staging System score (ISS equal to 1 or 2 or 3) [14], hematopoietic stem cell transplantation, date of diagnosis, number of treatment lines, prior IMID treatment, number of IMID cycles. Clinical and dispensing data were obtained from the medical and pharmaceutical software of our hospital.

### Statistical analysis

Questionnaire scores and MPR values were compared with a Kruskal–Wallis test for each IMID (lenalidomide, thalidomide, and pomalidomide). The measure of agreement between questionnaire scores and MPR values was assessed. First, we considered these variables as continuous variables and we used the Lin’s concordance correlation coefficient. Second, we considered these variables as categorical variables and we used the Cohen’s Kappa. The relationship between the questionnaire scores and variables of interest (MPR, demographic and clinical variables) was evaluated by multiple linear regression with a robust variance estimator. Missing data were taken into account using full information maximum likelihood (FIML). FIML estimation adjusts the likelihood function so that each case contributes information on the variables that are observed. A structural equation model was used assuming multivariate normality. Cronbach’s alpha (with an inter-item correlation matrix) was used to assess the reliability of the different questions. Item Response Theory with one parameter logistic model was used to explain the relationship between the questionnaire’s responses. Statistical analysis was performed using Stata (14.0, Stata Corporation, College Station, TX, USA). A p-value below 0.05 was considered significant. In our analyses, ISS 2 and ISS 3 variables corresponded to patients with ISS equal 2 and ISS equal 3.

## Results

### Patients

Sixty-three patients were included in our study. Patient characteristics are presented in Table 1. Lenalidomide was the most frequent IMID prescribed in our study (54%). Thirty-two patients (51%) had a previous treatment with another IMID or had been taking the same treatment for at least 6 months. The median number of IMID cycles was 4 (range 2–20).

**Table 1:** Patients characteristics (n = 63).

Characteristic	Measure	Result
**Gender**	N (%)	
• Male		42 (67%)
• Female		21 (33%)
**Age** (years)	Mean (SD)	68.7 (10.3)
**IMID**	N (%)	
• Lenalidomide		34 (54%)
• Pomalidomide		16 (25%)
• Thalidomide		13 (21%)
**International Staging System**	N (%)	
• Unknown		15 (24%)
• ISS 1		13 (21%)
• ISS 2		25 (40%)
• ISS 3		10 (16%)
**Hematopoietic Stem Cell Transplantation**	N (%)	31 (49%)
**Time since diagnosis** (days)	Median (range)	1034 (58–6235)
**Number of treatment lines**	Median (range)	2 (1–6)

### Adherence

Medication adherence for each IMID and each patient was estimated with the questionnaire score and the MPR (Table 2). The mean questionnaire score was 8.2±1.2, with the highest scores for lenalidomide, followed by thalidomide and then pomalidomide. The mean MPR was 0.97±0.06, with the highest for thalidomide, followed by lenalidomide and then pomalidomide. A total of 76% of patients were considered adherent according to the questionnaire (*i*.*e*. score ≥ 8), 94% according to the MPR (*i*.*e*. ≥ 0.90), and 70% according to the questionnaire and the MPR (*i*.*e*. questionnaire score ≥ 8 and MPR ≥ 0.90).

**Table 2:** Adherence results.

	Questionnaire score	p*	MPR	p*
**IMID**, mean (SD); median (min-max)		0.710		0.091
Lenalidomide	8.3 (1.0); 9 (6–10)		0.97 (0.06); 0.98 (0.76–1.09)	
Pomalidomide	7.9 (1.6); 8 (4–10)		0.96 (0.04); 0.98 (0.91–1.00)	
Thalidomide	8.2 (0.9); 8 (6–9)		1.01 (0.07); 1.00 (0.92–1.19)	
**Total**	8.2 (1.2); 8 (4–10)		0.97 (0.06); 0.98 (0.76–1.19)	

MPR, Medication Possession Ratio; SD, Standard Deviation.

*Kruskal-Wallis test

#### Questionnaire

The questionnaire results are detailed in Table 3. The mean time needed to complete the questionnaire was 9.2 ± 4.7 minutes. Fifty seven percent of the patients used tools to help them with their medicine intake. Twenty-seven percent had a caregiver (family or not) helping them with their medicine intake. A total of 70% had observed at least one adverse event with their treatment.

**Table 3:** Adherence questionnaire results.

Questions	Lenalidomide	Pomalidomide	Thalidomide	All
	Yes, N (%)	Yes, N (%)	Yes, N (%)	Yes, N (%)
1. This morning did you forget to take your medicine?	0	1 (6.3)	0	1 (1.6)
2. Since the last visit have you run out of medicine?	1 (2.9)	0	1 (7.7)	2 (3.2)
3. Do you ever take your medicine too late in comparison with usual time?	12 (35.3)	7 (43.8)	7 (53.8)	26 (41.3)
4. Sometimes if you feel worse when you take your medicine, do you stop taking it?	0	1 (6.3)	1 (7.7)	2 (3.2)
5. Do you think that you take too many medications? *	22 (64.7)	7 (43.8)	6 (46.2)	35 (56.5)
6. Do you ever not take your medicine because you forgot to do so?	7 (20.6)	4 (25.0)	0	11 (17.5)
7. Do you know the name of your medications?	21 (61.8)	8 (50.0)	7 (53.8)	36 (57.1)
8. Do you ever miss doses of your medicine when you feel sick?	1 (2.9)	2 (12.5)	2 (15.4)	5 (7.9)
9. Does a change in your daily routine modify the way you take your medicine?	1 (2.9)	3 (18.8)	0	4 (6.3)
10. Do you sometimes skip doses of your medicine when you feel better?	0	0	0	0 (0.0)

* n = 62 responses.

#### MPR

MPR values for each IMID are presented in Table 2. A total of 13 patients (21%) had at least one treatment discontinuation or dosage adjustment during the study period. The mean discontinuation time was 33 days (minimum 3 –maximum 106 days). The reasons for treatment discontinuation were either treatment-related adverse events (3 infections, 2 neutropenia, 1 anemia, 1 deep vein thrombosis) or other medical problems (2 vertebral osteosynthesis, 1 cholelithiasis).

### Concordance

No statistically significant agreement was observed between questionnaire scores and MPR values. The Lin’s concordance correlation coefficient was not significantly different from zero (p = 0.687). The Cohen’s Kappa was -0.1114 [95% CI, -0.1776 to 0.1055].

### Questionnaire analysis

The optimal number of factors introduced in the final multiple linear regression model was six: MPR, age, gender, number of treatment lines, ISS2 and ISS3 (Table 4). No statistically significant linear association was observed between the questionnaire score and these six factors. Gender and ISS were two factors with a p-value relatively near or below 0.2 (gender: β = 0.46; SD = 0.36; p = 0.206; ISS2: β = -0.56; SD = 0.32; p = 0.088).

**Table 4:** Multiple linear regression of adherence questionnaire score.

Variables	Coefficient β	Standard error	p
MPR	1.24	2.64	0.642
Age	0.01	0.01	0.434
Gender	0.46	0.36	0.206
Number of treatment lines	-0.01	0.18	0.977
ISS 2*	-0.56	0.32	0.088
ISS 3*	-0.70	0.54	0.200
Constant	6.36	2.96	0.036

*ISS 2 and ISS 3 are compared to ISS 1 (patients with ISS equal 1).

The reliability of the various questionnaire questions was calculated with an inter-item correlation matrix (Table 5). Some questions were unbalanced, in particular questions 1, 2, 4 and 10. All patients answered “no” to question 10, which was excluded from the reliability evaluation. Finally, all Cronbach’s alphas were relatively low (range 0.0342–0.2443), showing a low correlation with the questionnaire score.

**Table 5:** Reliability of adherence questions: Inter-item correlation matrix.

	Q1	Q2	Q3	Q4	Q5	Q6	Q7	Q8	Q9
Q1	0.0182								
Q2	-0.0003	0.0312							
Q3	0.0104	-0.0133	0.2463						
Q4	0.0000	0.0151	0.0028	0.0312					
Q5	0.0101	-0.0143	0.0346	0.0021	0.2499				
Q6	0.0152	0.0105	0.0558	0.0105	0.0034	0.1464			
Q7	-0.0101	0.0138	0.0184	-0.0023	-0.0040	-0.0046	0.2488		
Q8	0.0007	-0.0136	0.0010	-0.0297	0.0029	-0.0020	-0.0184	0.0742	
Q9	0.0003	0.0020	-0.0056	-0.0141	0.0122	-0.0049	-0.0115	0.0271	0.0604

Item Response Theory was used to explain the relationship between the responses of the questionnaire (Table 6). We observed a 75% likelihood that two respondents would provide the same answer. Questions 7 and 5 were those with the lowest coefficient (respectively -0.43312 [95% CI, -1.24319 to 0.37694] and -0.39633 [95% CI, -1.19219 to 0.39953]). Questions 9, 2, 4 and 1 were those with the highest coefficients (range 3.92072 to 5.59662).

**Table 6:** Item response theory.

	Coefficient	95% Confidence Interval	
Q7	-0.43312	-1.24319	0.37694
Q5	-0.39633	-1.19219	0.39953
Q3	0.53147	-0.19116	1.25410
Q6	2.30739	1.10509	3.50969
Q8	3.58537	1.55727	5.61348
Q9	3.92072	1.67673	6.16471
Q2	4.92218	1.78876	8.05560
Q4	4.92218	1.71872	8.12565
Q1	5.59662	2.40019	8.79305
Discrim	0.74625	0.38963	1.10287

## Discussion

To date, very few data are available about IMID adherence in MM patients. For different reasons such as cost, adverse events, long-term intake, or health status improvement, adherence to IMIDs may not be optimal. Our study is the first prospective study to evaluate adherence to IMIDs in the real life of MM patients.

The MPR is the most widely used indirect method for measuring adherence [12]. However, attempts to standardize its calculation have failed. For a rigorous investigation of the MPR, Sperber *et al*. have recently recommended displaying trends rather than static numbers, with the Fixed MPR trend as a lower limit and the Variable MPR trend as an upper limit [12]. The revised MPR used in the present study has a close definition of that of the Variable MPR. Our MPR took into account all discontinuation of treatment decided by a physician for medical reasons, and may thus be considered as accurate.

Our mean MPR value was 0.97±0.06. This value is higher than that of 0.85 reported in 6731 American MM patients between 2011 and 2013 [10]. Our mean MPR value was 1.01, 0.97, and 0.96 for thalidomide, lenalidomide, and pomalidomide, respectively. The authors of the American study reported a mean MPR value of 0.83, 0.85, and 0.91 for thalidomide, lenalidomide, and pomalidomide, respectively [10]. We may hypothesize that the difference is due to the fact that American data were only obtained retrospectively from pharmacy claims without analysis of treatment discontinuation for medical reasons.

The mean questionnaire score was 8.2, close to that of 8.7 reported in CML patients treated with imatinib [13]. The questionnaire score was the lowest for pomalidomide (mean value 7.9). Pomalidomide is for now only used in France in patients with at least two prior treatment lines including bortezomib and lenalidomide. These patients may have advanced disease-related symptoms and they also may present cumulative toxicities (in particular neurological). This has been confirmed in an observational study that described prescribing practices of pomalidomide in a cohort of 63 consecutive MM patients. After a median follow-up of 28 months, 6% of patients had discontinued pomalidomide following severe adverse events, and 38% of patients had required a dose decrease following adverse events [15].

Our adherence results are relatively high in comparison with the results between 40 and 100% reported in the review by Bassan *et al*. for oral anticancer agents [7]. Due to the severity of the disease, patients with cancer are mostly intensively implicated in their management and especially in the intake of anticancer agents [16]. Moreover, in France, MM patients do not pay for IMIDs. In countries like the US, patients with cancer have an increased risk of treatment-related financial toxicity because of rising treatment costs and prolonged duration of treatment. In 100 US patients, 46% used savings to pay for MM treatment, and 21% borrowed money to pay for medicines [17]. The high adherence in our study may be explained by intensive medical follow-up with scheduled visits every 4 weeks. Furthermore, the daily presence of a clinical pharmacist in the ward may contribute to this elevated adherence. Ribed *et al*. showed that with the intervention of a clinical pharmacist (three clinical interviews focused on safety and efficiency outcomes), the number of adherent patients (defined with a MPR>90%) increased by 20% at six months in the intervention group (p<0.001) [18]. The high percentage of adherence may also have been influenced by the presence of caregivers or family members. However, their influence is very difficult to evaluate. The percentage of patients in our study who required help for IMIDs intake was close to that of 31% reported by Arber *et al*. in a study of MM patients [19].

Using a specific questionnaire that had never been used in MM patients, our objective was also to explore a new method to evaluate adherence. The development and validation of (self)-assessment adherence tools are of major interest whatever the therapeutic area. Questionnaires are easy-to-use tools for health care professionals and/or patients, and they give health care professionals the opportunity to assess adherence during routine clinical practice and to suggest targeted interventions. Questionnaires have the key advantage of being able to rapidly identify potentially non-adherent patients. For the present study, we used a previously developed specific adherence questionnaire for CML patients receiving imatinib that was created using questions from several other questionnaires [20–22]. This decision was made for the following reasons: CML and MM are two hematological malignancies; imatinib and IMIDs are oral anticancer agents; imatinib and IMIDs are the cornerstone of treatment for the two diseases; and, finally, the questionnaire has been validated in CML patients to evaluate imatinib adherence. For questions 5 and 7, we observed the same findings as for imatinib in CML patients. With item to total correlation coefficients below 0.2 in the study by Daouphars *et al*., and with lowest item response theory coefficients in our study, these two questions seem to explore other dimensions than just adherence [13]. In our study, the concordance of the two methods for measuring the adherence was null. Considering MPR as the “gold standard” method for measuring adherence, the questionnaire does not give reproducible results. As a result, the questionnaire should be used with caution to evaluate IMID adherence.

No statistically significant association was shown between the questionnaire scores and MPR values in our study, whether considering variables as continuous or categorical, which is not necessarily surprising. MPR is calculated from administrative data while the questionnaire score is calculated from data orally given by patients. The MPR explicitly recognizes that adherence measured with claims data provides evidence for receiving a drug, but no evidence that it has been used. Moreover, the phenomenon of unnecessary drug storage is very well known [23]. Though questionnaires have a major declaration bias, each method for evaluating adherence has strengths and weaknesses. Each method probably provides complementary information. In the context of limited resources, easy-to-use questionnaires may be useful for the rapid identification of non-adherent patients.

Our study has some limitations. First, our study is monocentric. Second, we included only 63 patients. On the other hand, we included consecutive patients without any exclusion criteria, therefore reflecting real life. We were able to do complete patient follow-up (no follow-up loss) and no adherence data were missing. With the use of a revised MPR, we consider that our adherence estimates are of high quality. Finally, the 63-patient sample size is quite small and may explain the lack of association in our linear regression model, even though we took into account Harrell’s rule to define the optimal number of variables to be introduced into the final model.

To conclude, our measures of adherence are relatively high with a total of 76% of patients considered as adherent according to the questionnaire, 94% according to the MPR and 70% according to the questionnaire and the MPR. These findings are encouraging in view of the efficacy, toxicity and elevated cost of IMIDs.

## Supporting information

S1 File Adherence results. (XLS)
